# Molecular Action of Hydroxytyrosol in Wound Healing: An In Vitro Evidence-Based Review

**DOI:** 10.3390/biom10101397

**Published:** 2020-09-30

**Authors:** Nike Dewi Utami, Abid Nordin, Haliza Katas, Ruszymah Bt Hj Idrus, Mh Busra Fauzi

**Affiliations:** 1 Centre for Tissue Engineering and Regenerative Medicine, Faculty of Medicine, Universiti Kebangsaan Malaysia, Cheras, Kuala Lumpur 56000, Malaysia; nike.dewiutami@gmail.com (N.D.U.); ruszyidrus@gmail.com (R.B.H.I.); 2 Department of Physiology, Faculty of Medicine, Universiti Kebangsaan Malaysia, Cheras, Kuala Lumpur 56000, Malaysia; m.abid.nordin@gmail.com; 3 Centre for Drug Delivery Research, Faculty of Pharmacy, Universiti Kebangsaan Malaysia, Jalan Raja Muda Abdul Aziz, Kuala Lumpur 50300, Malaysia; haliza.katas@ukm.edu.my

**Keywords:** hydroxytyrosol, wound healing, cellular, antioxidant, anti-inflammatory, antimicrobial

## Abstract

Hydroxytyrosol (HT) is an essential molecule isolated from the phenolic fraction of olive (*Olea europaea*). HT has been implicated for its health-stimulating effect mainly due to its antioxidative capacity. The current review summarises and discusses the available evidence, related to HT activities in wound healing enhancement. The literature search of related articles published within the year 2010 to 2020 was conducted using Medline via Ebscohost, Scopus, and Google Scholar databases. Studies were limited to in vitro research regarding the role of HT in wound closure, including anti-inflammation, antimicrobial, antioxidative, and its direct effect to the cells involved in wound healing. The literature search revealed 7136 potentially relevant records were obtained from the database search. Through the screening process, 13 relevant in vitro studies investigating the role of HT in wound repair were included. The included studies reported a proangiogenic, antioxidative, antiaging, anti-inflammatory and antimicrobial effect of HT. The current in vitro evidence-based review highlights the cellular and molecular action of HT in influencing positive outcomes toward wound healing. Based on this evidence, HT is a highly recommended bioactive compound to be used as a pharmaceutical product for wound care applications.

## 1. Introduction

### 1.1. Wound Healing

A wound can be defined as a pathological condition involving the devastation of normal anatomic tissue microstructure and function. It is the consequence of chemical, thermal or physical injury leading to an inflammatory response from the penetration of foreign materials and organisms into the damaged tissue [1,2].

A ‘wound’ can be classified into acute and chronic based on the wound closure duration. Acute wounds like burns, traumatic injuries and non-infected surgically created wounds, take approximately 8–12 weeks to be repaired. On the contrary, chronic wounds may need more than 12 weeks to be closed, such as diabetic foot ulcers, venous leg ulcers and pressure ulcers [3,4,5].

The reduction of wound area is a dynamic biological process, triggered by ruptured tissue, with overlapping multicellular phenomenon involving numerous elements, such as platelets, leukocytes, monocytes, neutrophils, macrophages, fibroblasts, keratinocytes, endothelial and transient myofibroblasts, to maintain tissue integrity [6,7,8,9]. The progression of wound healing is divided into four sequential phases, including haemostasis, inflammatory, proliferative and remodelling [10].

Haemostasis phase involves three main processes, including platelets activation, vasoconstriction and leukocytes activation to the injury site [7]. The inflammatory stage is characterised by vasodilatation followed by neutrophils infiltration, monocytes activation and macrophages penetration into the wound site [8]. Prolonged inflammation may halt the progression of wound healing into the subsequent proliferative and remodelling phase [11].

Furthermore, the proliferative phase comprises fibroplasia, re-epithelialisation and angiogenesis. At this stage, several cells begin to proliferate and migrate to the defected area to achieve complete wound closure [9]. Lastly, tissue remodelling phase is the final stage in wound healing, whereby maintenance of balance between the degradation and the synthesis of the ECM continues until a fully restored tissue structured is achieved [12].

### 1.2. Molecular Mechanism of Wound Healing

#### 1.2.1. Anti-inflammatory Activity

As previously mentioned, prolonged inflammation has been attributed to a delay in wound healing. In normal wound, inflammation subsides with the decreased production of pro-inflammatory cytokines and action of anti-inflammatory cytokines [11]. In non-healing wound, pro-inflammatory cytokine was found to be higher compared to acute wounds [13]. Many of the cytokines were regulated via the nuclear factor kappa B (NF-κB), a transcription factor that resides in the cytoplasm and translocates to the nucleus when activated. Once in the nucleus, NF-κB can activate the transcription of a plethora of cytokines [14].

On the other hand, matrix metalloproteinases (MMPs) were also found to be abundant in a non-healing wound [13]. MMPs are extracellular proteases secreted at wound sites that have the ability to degrade growth factors and ECM composition. MMPs also can influence the inflammation process as soluble factors with both pro and anti-inflammatory effect [15]. Therefore, inflammatory cytokines, NF-κB and MMPs can be used as indicators to predict the anti-inflammatory activity and the progression of wound closure.

#### 1.2.2. Antimicrobial Activity

A paramount reason for having the inflammatory phase is to clear foreign materials, including bacteria from the wound environment [8]. Chronic infections can also lead to the prolongation of the inflammatory state. Hence, an antimicrobial agent is the utmost importance in ensuring the progression of the normal wound healing process [16].

In chronic wounds, the oxygen concentration in tissue is low, due to low blood flow and high oxygen consumption [17]. The lack of oxygen or hypoxic condition is well-related to the increment of bacterial colonisation. including *Staphylococcus* species, *Streptococcus* species, *Pseudomonas aeruginosa* and *Escherichia coli*, which are dominant microorganisms found in wound site [18,19,20]. Besides, oxidative stress environment in non-healing wounds promotes extracellular matrix (ECM) destruction that supplies nutrition for the microbes [21]. Hence, an antioxidant could have a beneficial effect on alleviating bacterial infection, which in turn, allows the normal progression of wound healing.

Effectiveness of an antimicrobial agent is indicated by two values that can be obtained through bacterial culture experiments. They are minimum inhibitory concentration (MIC) and minimum bactericidal concentration (MBC) [22]. The MIC is defined as the minimal concentration of antibiotic that prevents a clear suspension of 10^5^ colony-forming units (CFUs) of bacteria/mL from becoming turbid after overnight incubation. While MBC demonstrates the lowest level of antimicrobial agent needed that results in microbial death. The MBC is identified by determining the lowest concentration of antibacterial agent that reduces the viability of the initial bacterial inoculum by ≥99.9%.

#### 1.2.3. Antioxidant Activity

Considering the oxidative stress reported in chronic wounds, an antioxidant agent should be beneficial in the progression of wound healing. Oxidative stress is the result of an imbalance between high levels of reactive oxygen species (ROS) formation and low level of antioxidative activity [23]. ROS is a short-lived and highly reactive molecule, in free radical form, secreted by neutrophils and macrophages immediately after an injury [24]. It has the ability to terminate bacterial survival, induce tissue destruction and inhibit cellular functions, such as cell proliferation and cell migration [25].

In addition, ROS production can be regulated through nuclear factor erythroid-2-related factor 2 (NRF-2) and heme-oxygenase 1 (HO-1) activation. NRF-2 is a helix-loop-helix basic leucine zipper transcription factor and a master regulator of cell responses to oxidative stress [26]. Activation of NRF-2 led to the production of HO-1, the rate-limiting enzyme that converted heme into carbon monoxide (CO) and biliverdin [27]. HO-1 significantly elevated in chronic wounds as protection against oxidative stress. Taken together, ROS, NRF-2 and HO-1 are good biomarkers to examine the antioxidant property and the progression of wound healing.

#### 1.2.4. Cellular Events

Cell proliferation and migration are critical cellular events in the proliferative phase of wound healing. Dermal fibroblasts, epithelial cells and endothelial cells are the three main cellular components in the regeneration of the human skin. Upon clearance of inflammatory cells, fibroblast migrates to the wound site and immediately degrade the loose provisional fibrin matrix left by the inflammatory cells [11]. As the proliferative phase progresses, fibroblasts proliferate and migrate to form the granulation tissue covered with a newly synthesised matrix. This is followed by the re-epithelialisation of the wound by the epithelial cells, migrating over the new matrix laid out by the fibroblasts [6,28,29]. Formation of a new blood vessel by the endothelial cells occur concurrently throughout all the proliferative phase [30]. Therefore, the proliferation and migration of dermal fibroblasts, epithelial cells and endothelial cells are suitable to be used as parameters for wound healing evaluation.

### 1.3. Hydroxytyrosol

Among all phenolic constituents of olives, hydroxytyrosol (HT) is currently being an attractive molecule to be utilised in the pharmaceutical, medical, nutraceutical, cosmeceutical and food industry. HT or 4-(2-hydroxyethyl)-1,2-benzenediol is a polar secondary plant metabolite, obtained from olives, derived from oleuropein through enzymatic hydrolysis. It has a typical catechol structure with chemical formula C_8_H_10_O_3_ and molecular weight of 154.16 g/mol [31].

## 2. Materials and Methods

### 2.1. Research Articles Search Strategy

A systematic review of the literature was conducted to identify relevant studies reporting the role of HT in wound healing. The Medline via Ebscohost (Ipswich, MA, USA), Scopus (Amsterdam, The Netherlands) and Google Scholar (Mountain View, CA, USA) databases were used to identify the relevant articles established between 2010 and 2020. The search strategy involved a combination of the following keywords: hydroxytyrosol* AND wound* OR acute* OR chronic*.

### 2.2. Selection of Research Articles

The results were limited only to the relevant articles published in the English language, due to limited resources for translation services. The research articles which primarily investigated the role of HT in wound care were included. Review articles, news, letter, editorials or case studies were excluded from the review. Research articles that were not in vitro experiments that investigated HT activity in promoting wound healing were removed.

### 2.3. Inclusion and Exclusion Criteria

For this evidence-based review, only in vitro studies that investigated the role of HT in wound healing were included. In vitro investigations included in this review must evaluate the role of HT in cellular events (cell proliferation, cell migration and cell viability) that are well-related to the enhancement of wound healing. Moreover, in vitro research examining the antioxidant, anti-inflammatory and antimicrobial property of HT, which can accelerate wound closure were also included. Studies that measure the changes in reactive oxygen species (ROS), nuclear factor erythroid-2-related factor 2 (NRF2), heme oxygenase-1 (HO-1), inflammatory cytokines (TNF-α, IL-1β, Il-6 and IL-8), nuclear factor kappa B (NF-κB), matrix metalloproteinases (MMPs) and transforming growth factor-beta (TGF-β), whenever available, were included. For this evidence-based review, all in vivo experiments investigated the role of HT in wound healing are excluded.

### 2.4. Data Extraction and Management

Articles were screened in three phases prior to their inclusion and exclusion criteria in this systematic review. The first phase; the titles and abstracts were screened to ensure inclusion and exclusion criteria adhered to. Second phase; abstracts of the remaining articles were screened, and abstracts that did not meet the inclusion criteria were excluded. In the final phase, the remaining articles were read thoroughly by four independent reviewers to exclude any articles that did not meet the inclusion criteria followed by data extraction. The following data were recorded from the studies: [1] The types of study; [2] aim of study; [3] subject or sample; [4] methodology or parameters; [5] results; and [6] conclusion.

## 3. Results

### 3.1. Literature Search

**Figure 1:**
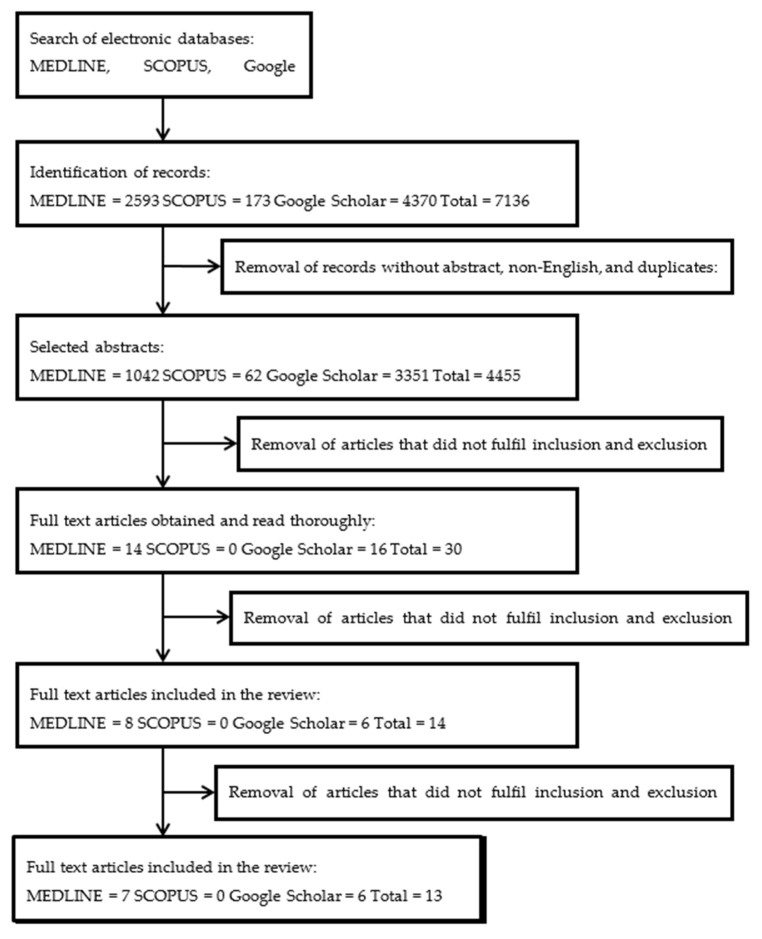
Flow chart of the selection process.

The extensive literature search successfully identified 7136 potentially relevant records. Initial screening of records resulted in the removal of 2681 records that were not the original article, not published in English language and a duplication. The articles were then screened based on the title for any inclusion criteria that resulted in the removal of 4425 articles. From the remaining 30 articles, 16 articles were removed after screening the abstracts for inclusion and exclusion criteria. Reviewers then read the full text of the remaining 14 papers, of which 1 article was excluded because they did not fulfil the inclusion criteria. Thirteen articles were finalised for data extraction. A flow chart of the article’s retrieval process was shown in Figure 1.

### 3.2. Study Characteristics

The summary of studies that examined the effect of HT in wound healing is displayed in Table 1. All studies were published within the year 2010 until 2020. Three studies reported the antiangiogenic effect of HT [32,33,34], while another three studies reported its proangiogenic effect [35,36,37]. Three studies examined the antioxidant effect of HT [38,39,40]. One study reported the antiaging effect of HT [41]. Three studies focused on investigating the antimicrobial effect of HT [42,43,44]. Moreover, eight studies also included the anti-inflammatory effect of HT as a supplementary outcome of their studies [32,33,34,35,38,39,40,41].

In terms of the experimental model, endothelial cells were used to evaluate the effect of HT on the angiogenesis process that is crucial in the proliferative phase of wound healing. Five studies utilised human umbilical vascular endothelial cell (HUVEC) in their studies, while two studies by Zrelli et al. (2015, 2011) [36,37] utilised porcine endothelial cells. For the antioxidative studies, one study each utilised human keratinocyte and monocyte, while another one study had both human keratinocyte and dermal fibroblast. HT antiaging effect was investigated in dermal fibroblast. Regarding antimicrobial effect, HT was tested against *Staphylococcus aureus* and *Staphylococcus epidermidis* by Ghalandari et al. (2018), against 12 variety of gram-positive and gram-negative bacteria by Medina-Martinez et al. (2016), and against *S. epidermidis* by Crisante et al. (2015).

### 3.3. Role of HT in Angiogenesis

Formation of new vasculature network within the injured tissue is crucial in supporting its restoration back to its functional state. Thus, the angiogenic property of hydroxytyrosol has become a subject of interest to 6 out of the 13 studies included [32,33,34,35,36,37]. Contradicting results were reported whereby three studies reported antiangiogenic properties with HT, while the remaining three studies reported proangiogenic properties of HT.

Chronologically, the proangiogenic properties of HT were reported first before its antiangiogenic properties. Zrelli et al. (2011) first reported the enhancement of cell proliferation and migration in their porcine vascular endothelial cells. HT also demonstrated the antioxidative effect by reducing the formation of ROS. At the molecular level, they found that the effect exerted by HT can be attributed to the upregulation of NRF-2 and its subsequent effector, the HO-1 [37]. In the follow-up study by the same group, they succeeded in elucidating the involvement of PI3K/Akt and ERK1/2 signalling pathways in the proangiogenic effect of HT on porcine vascular endothelial cells [36].

Various forms of electrical stimulation, including direct current, combined electromagnetic fields and pulse electromagnetic field (PEMF), have been demonstrated to positively influence the process of angiogenesis [45]. Correspondingly, Cheng et al. (2017) investigated the effect of combining HT and PEMF on HUVEC proliferation and migration. Indeed, PEMF alone demonstrated enhancement of HUVEC proliferation and migration. HT addition enhances these effects on HUVEC [35].

Endothelial dysfunction and impaired angiogenesis during diabetes have been attributed as one of the contributors to a chronic non-healing wound in diabetic patients [46]. More recent studies by Cerezo et al. (2019), Calabriso et al. (2018) and Lopez et al. (2017) revealed an antiangiogenic property of HT in HUVEC [32,33,34]. Angiogenesis was triggered by TNF-α by Lopez et al. (2017) [34], phorbol myristate acetate (PMA) by Calabriso et al. (2019) [33] and vascular endothelial growth factor (VEGF) by Cerezo et al. (2019) [32]. In all three studies, HT exerts a regenerative effect on the HUVEC via suppression of oxidative stress, inflammation, cell adhesion and cell migration.

### 3.4. Role of HT in Oxidative Stress

Oxidative stress in wound microenvironment is the imbalance condition of reactive oxygen species (ROS) concentration and the ability of biological systems to rebuild the resulting damage [47]. Thus, the antioxidant property is essential to improve wound healing. Avola et al. (2018), Meschini et al. (2018), and Guo et al. (2010) have investigated the antioxidant action of HT [38,39,40].

The earliest study focusing on the antioxidative effect of HT in the context of wound healing is by Guo et al. (2010) [40]. By utilizing the epidermal keratinocyte cell line, HaCat cells, they investigated the effect of HT on alleviating oxidative stress caused by UVB light. As expected, HT suppresses the oxidative stress caused by UVB light on HaCat cells indicated by the significant decrease in the detection of oxidised dichloro-dihydro-fluorescein diacetate (DCFH-DA) and 8-hydroxy-2′-deoxyguanosine (8-OHdG) within the cell.

In another study, Avola et al. (2018) utilised both keratinocytes and dermal fibroblasts to investigate the antioxidative effect of HT caused by light-emitting-diode-generated blue light (LED-BL) irradiation [38]. As anticipated, HT suppresses the oxidative stress caused by LED-BL on both keratinocytes and dermal fibroblasts indicated by the significant decrease in the detection of oxidised DCFH-DA and 8-OhdG within the cell.

HT antioxidative effect had also been investigated in the inflammatory model of lipopolysaccharide (LPS)-stimulated human THP-1 monocytes. In this study, Meschini et al. (2018) investigated enzyme-treated olive vegetation waste (OVW) instead of synthesised HT [39]. They were able to demonstrate a significantly higher yield of HT in enzyme-treated OVW when measured with gas chromatography mass spectrometry (GC-MS) compared to the non-treated OVW. When evaluated for antioxidative effect, all OVW were able to reduce the 2,2-diphenyl picrylhydrazyl (DPPH) activity. However, the enzyme-treated OVW shown significantly greater antioxidative effect compared to the non-enzyme-treated OVW. This suggested that a higher content of HT resulted in a greater antioxidative effect.

### 3.5. Antiaging Properties of HT

Skin aging can influence wound healing capabilities by causing decreased proliferative responses, delayed angiogenesis, delayed remodelling, and slower re-epithelialisation [48]. In dermal fibroblast, declined motility, proliferative capacity and chemotactic response can be observed with aged-related cellular defects [49]. Consequently, Jeon and Choi (2018) utilised dermal fibroblast to evaluate the antiaging effect of HT following photoaging induced by UVA light [41]. By measuring the senescence-associated β-galactosidase (SA-β-gal) in the dermal fibroblast, they found that HT treatment significantly reduces the number of cells presented with SA-β-gal, suggesting the antiaging effect of HT.

### 3.6. Antimicrobial Properties of HT

In terms of antimicrobial activity, three studies revealed that HT possesses the ability to inhibit bacterial growth. Studies conducted by Ghalandari et al. (2018) unravelled antimicrobial activity against *S. aureus* and *S. epidermidis* [42]. The minimum inhibitory concentration (MIC) and minimum bactericidal concentration (MBC) value of HT on *S. aureus* were 3.125 and 6.25 mg/mL. Meanwhile, the MIC and MBC values of HT on *S. epidermidis* were 6.25 and 12.5 mg/mL. The inhibition zone of HT (3.125; 6.25; 12.5; 25; 50 and 100 mg/mL) on *S. aureus* were 16, 19, 22, 24, 27 and 30 mm. The inhibition zone of HT in the same range of concentrations on *S. epidermidis* were 11, 13, 16, 19, 22 and 25 mm.

In addition, Medina-Martinez et al. (2016) evaluated the effect of HT against 12 different bacterial species [43]. They included eight g-negative bacteria, namely, *Erwinia carotovora*, *Klebsiella pneumonia*, *Pseudomonas aeruginosa*, *Yersinia enterocolitica*, *Salmonella thypimurium*, *Aeromonas hydrophila*, *Shigella sonnei*, *Escherichia coli* and 4 g-positive bacteria, namely, *Pediococcus acidilactici*, *Kocuria rhizophila*, *Listeria monocytogenes*, and *S. aureus*. Generally, HT exhibited MIC of 1000 μg/mL against most bacteria except 400 µg/mL MIC against *S. aureus* and *E. coli*. They furthered their investigation using four different strains of E. *coli* and concluded that 200 and 400 of HT μg/mL significantly reduced bacterial growth rate compared to the control, while 1000 μg/mL dose resulted in complete inhibition of growth.

Finally, Crisante et al. (2015), demonstrated that their HT containing polyacrylate was able to reduce the growth of *S. epidermidis* with inhibition zone 10 mm at 10 mg/mL and MIC value 2.5 mg/mL [44].

### 3.7. Role of HT in Inflammation

Excessive levels of inflammatory cytokines, such as TNF-α, IL-6, IL-8 and IL-1β, have been implicated in delaying wound healing [50]. Therefore, anti-inflammatory agent is required to alleviate the inflammatory state and allow progression of wound healing. Inflammatory cytokines were evaluated in eight studies, complementary to the angiogenesis, antioxidative, and antiaging parameters.

TGF-β1 has been implicated in promoting angiogenesis among its other functions. Thus, Cheng et al. (2017) decided to investigate the TGF-β1 expression and its underlying molecular mechanism [35]. In their PEMF induced HUVEC, significant upregulation of TGF-β1 compared to the untreated HUVEC was observed. This upregulation was amplified with the treatment of HT. This regulation of TGF-β1 by PEMF and HT was found to be mediated by the Akt and mTOR signalling pathways.

During inflammation, the nuclear factor κβ (NF-κβ) initiate the transcription of many cytokines gene [14]. Therefore, the inhibition of NF-κβ has become a target in controlling inflammatory cytokines. HT has been shown to suppress the upregulation of NF-κB and p53 in HaCat cells. Lopez et al. (2017) went further to investigate the effect of HT on the regulators of NF-κβ, nuclear factor of kappa light polypeptide gene enhancer in B-cells inhibitor, alpha (IκBα) and inhibitor of nuclear factor kappa-B kinase subunit beta (IKKβ) [34]. In their HUVEC, HT suppresses the TNF-α induced activity of IκBα and IKKβ. This will, in turn, shut down the NF-κβ signalling pathway and cease the production of inflammatory cytokine as evidence by the downregulation of the chemokine (CAC) motif ligand 2 (CCL2), prostaglandin-endoperoxidase synthase 2 (PTGS2).

Tumour necrosis factor α (TNF-α) is the pro-inflammatory cytokines that are responsible for a diverse range of signalling events during inflammation, leading to necrosis or apoptosis of target cells [51]. Exacerbation of TNF-α has been implicated in delayed wound healing. Hence, suppressing its production can serve as beneficial to wound healing progression. Calabriso et al. (2018) managed to show the TNF-α suppression by HT in their PMA-induced HUVEC [33]. The TNF-α suppressing effect of HT was also observed in LPS-induced THP-1 monocyte.

Suppression of interleukins (Ils) by HT was also reported in the studies included. In terms of IL-1β, HT has been shown to suppress its upregulation in PMA-induced HUVEC, TNF-α-induced THP-1 monocyte, and UVA-induced dermal fibroblast. Other Ils, such as IL-6 and IL-8, were also investigated. HT displays suppression of IL-6 in TNF-α-induced THP-1 monocyte and UVA-induced dermal fibroblast. IL-8 was found to be suppresses in UVA-induced dermal fibroblast.

## 4. Discussion

In this in-vitro evidence-based review, we summarised findings from 13 experiments evaluating the role of hydroxytyrosol (HT) in wound healing. Among factors that can impede wound repair are oxidative stress, abnormal angiogenesis, persistent inflammation and microbial colonisation. The evidence supported HT’s high potential as a bioactive phenolic molecule isolated from olives to prevent the abovementioned factors, due to its powerful antioxidant, angiogenesis modulation, anti-inflammatory and antibacterial activities.

Furthermore, the studies included employs a variety of cellular model that are involved in different phases of wound healing. This enables the understanding of HT role throughout the process of wound healing. Starting at the inflammatory phases, HT exerts inhibitory effect that may allow progression of wound healing into the proliferation phase. At the proliferation phase, HT displays positive effects on the granulation tissue formation, angiogenesis and re-epithelialisation process.

Regarding antioxidative effect of HT, the three studies included were limited to the measurement of ROS production and suppression. Oxidative stress can also be triggered by reduced activity of the endogenous antioxidant enzymes, such as superoxide dismutase (SOD), catalase (CAT), glutathione peroxidase (GPx), glutathione reductase (GRx), and glutathione-S-transferase (GST) [24]. Assessment of the effect of HT on these enzymes can provide a wholesome picture of the role of HT in modulating oxidative stress in injured tissue.

Aging has long been associated with oxidative stress [52]. Although SA-β-gal in dermal fibroblasts indicates cellular aging, it can be interpreted too as the accumulation of oxidative stress. Similar to Avola et al. (2018) and Guo et al. (2010), Jeon and Choi (2018) also challenged their cells with irradiation of a specific spectrum of light [38,40,41]. It can be hypothesised that, if Jeon and Choi (2018) evaluated ROS, they would also find the antioxidative effect of HT [41]. Alternatively, if Avola et al. (2018) and Guo et al. (2010) measured antiaging parameters, they would also find the antiaging properties of HT [38,40].

**Figure 2:**
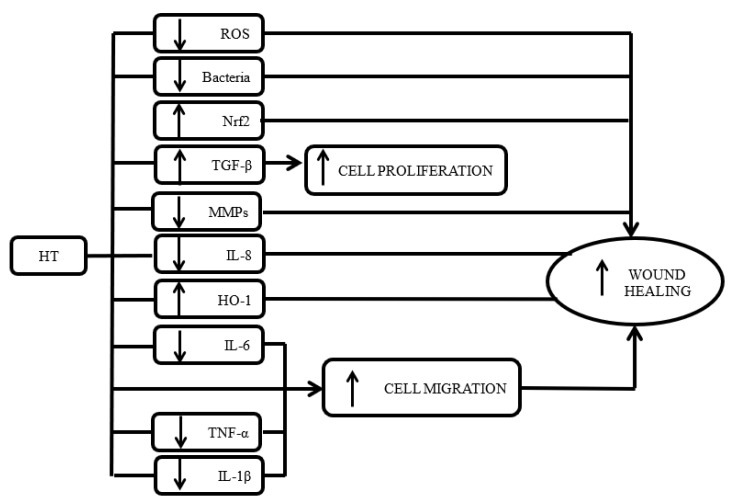
Illustration of the molecular action of HT in wound healing.

Within the context of inflammation, an assortment of inflammatory mediators used by the studies included enabled the mapping of HT effect on the signalling network of inflammation. Figure 2 illustrates the effect of HT on different cytokines. This can serve as a guide for the therapeutic use of HT in modulating inflammation. Moreover, results from different studies, employing different cell types, were similar according to the cytokines. This suggests a consistent effect of HT across different cells involved in wound healing. However, these data have to be considered with caution as the number of published studies is not large enough to draw a consensus.

## 5. Conclusions

This in vitro evidence-based review provided data that support the potential of HT as a bioactive compound to enhance wound reduction, due to its antioxidant, angiogenesis modulation, anti-inflammatory and antibacterial activities. The information in this review could potentially be applied to various current technology, including tissue engineering, drug delivery system and cell-free treatment that benefit different unresolved situation, such as chronic wound healing, severe ulcer, burn, postsurgical wound, and others.

## Figures and Tables

**Table 1:** Effect of hydroxytyrosol (HT) in wound healing.

Antiangiogenic Properties of HT					
No.	References	Aim of Study	Subject/Treatment	Parameters	Results
1.	Cerezo et al. 2019 [32]	To investigate the effects of serotonin, melatonin, 3-indoleacetic acid, 5-hydroxytryptophol and hydroxytyrosol (HT) on vascular endothelial growth factor (VEGF) activity.	Subject Human umbilical vein endothelial cells (HUVECs) induced with VEGF (25 ng/mL) for 5, 10 or 60 min. Treatment groups 1 µM of serotonin, melatonin, 3-indoleacetic acid, and 5-hydroxytryptophol each, as well as 50 µM of HT.	1. Cell migration. 2. VEGF receptor 2 (VEGFR-2) phosphorylation. 3. PLCγ1, Akt and eNOS phosphorylation.	1. Serotonin and 5-hydroxytryptophol inhibited HUVEC migration by 97% and 50%, respectively. No result for melatonin and HT were reported. 2. All of the test compounds, whether alone or in combination, inhibits VEGFR-2 phosphorylation. 3. Only HT inhibits PLCγ1, while HT, melatonin and serotonin shared enhancement effect on Akt and eNOS.
2.	Calabriso et al. 2018 [33]	To investigate the HT effects on endothelial dysfunction under inflammatory conditions.	Subject HUVECs induced with phorbol myristate acetate (PMA; 10 nmol/L). Treatment groups HT at 0, 1, 10, 30 µmol/L concentrations.	1. mRNA levels of tumour necrosis factor-α (TNF-α) and interleukin-β (IL-1β). 2. mRNA levels of intercellular adhesion molecule-1 (ICAM-1), vascular adhesion molecule-1 (VCAM-1). 3. HUVEC cell migration. 4. HUVEC tube formation. 5. Mitochondrial and cytosolic reactive oxygen species (ROS) production. 6. Superoxide dismustase (SOD) activity and malondialdehyde (MDA) level. 7. Mitochodrial membrane potential. 8. Mitochondrial ATP synthase activity. 9. Protein and mRNA levels of ATP5β. 10. Protein and mRNA levels of peroxisome proliferator-activated receptor gamma coactivator 1-alpha (PGC-1α), nuclear respiratory factor-1 (NRF-1), and mitochondrial transcription factor A (TFAM).	1. HT attenuates PMA-induced upregulation of TNF-α and IL-1β. 2. HT attenuates PMA-induced upregulation of ICAM-1 and VCAM-1. 3. HT inhibits PMA-induced migration of HUVECs. 4. HT inhibits PMA-induced tube formation by HUVECs. 5. HT attenuates PMA-induced elevation of mitochondrial and cytosolic ROS level. 6. HT suppresses PMA-induced elevation of SOD activity and MDA level. 7. HT restores the PMA-induced depolarisation of the mitochondrial membrane potential. 8. HT restores the PMA-induced reduction of ATP synthase activity. 9. HT restores the PMA-induced downregulation of ATP5β. 10. HT restores the PMA-induced downregulation of PGC-1α and NRF-1, as well as the upregulation of TFAM.
3.	Lopez et al. 2017 [34]	To investigate the effects of chemically synthesised metabolites (sulphate and glucuronate forms) from HT on oxidative stress and inflammation in TNF-α activated HUVECs.	Subject HUVECs induced with TNF-α. Treatment groups HT, HT sulfonate (HT-SUL), HT glucuronate (HT-GLU) at 0-200 µM concentrations.	1. HUVEC cell viability. 2. ROS production Glutathione (GSH) level. 3. Gene expression of antioxidant enzymes glutathione peroxidase 1 (GPX1) and glutamate-cysteine ligase catalytic subunit (GCLC). 4. Gene and protein expression of HO-1. 5. Phosphorylation of IKKαβ, IκBα, and p65. 6. Gene and protein expression of adhesion molecules ICAM-1, VCAM-1, and E-selectin. 7. mRNA levels of, chemokine (CAC) motif ligand 2 (CCL2), prostaglandin-endoperoxidase synthase 2 (PTGS2).	1. HUVEC cell viability was not affected up to 100 µM concentrations of HT and its metabolite. 2. HT and HT-SUL suppress TNF-α-induced ROS formation. 3. HT and HT-SUL attenuates TNF-α suppression of GSH. 4. HT and HT-SUL attenuates TNF-α induced downregulation of GPX1 and GCLC. 5. HT and HT-SUL attenuates TNF-α induced downregulation of HO-1. 6. HT and all metabolites attenuate TNF-α induced phosphorylation of IKKαβ, IκBα, and p65. 7. HT and all metabolites suppress TNF-α induced upregulation of ICAM-1, VCAM-1 and E-selectin. 8. HT and all metabolites suppress TNF-α induced upregulation of CCL2 and PTGS2.
4.	Cheng et al. 2017 [35]	To analyse the effects of HT on the proliferation and differentiation of human umbilical vein endothelial cells (HUVECs).	Subject HUVECs exposed to pulsed electromagnetic fields (PEMFs). Treatment groups HT at 0, 10, 30, 50, 100 and 150 μM concentrations.	1. Cell proliferation. 2. Cell migration. 3. mRNA and protein levels of Akt, mTOR, TGF-β1, and p53.	1. HT enhances PEMF-induced HUVEC proliferation with 30 μM concentration resulted in the highest cell proliferation. 2. HT enhances PEMF-induced HUVEC cell migration compared with the control and PEMF treatment only. 3. Combination of HT and PEMF treatment elevated the mRNA and protein levels of Akt, mTOR and TGF-β1, but not p53 when compared to the control.
5.	Zrelli et al. 2015 [36]	To evaluate the expression of HO-1 and NRF-2 in HT-induced endothelial wound healing.	Subject Porcine vascular endothelial cells (VECs). Treatment groups HT at 10, 30, 50 and 100 μM concentrations.	1. mRNA levels of heme oxygenase 1 (HO-1). 2. Protein levels of HO-1 and NRF-2 following inhibition of PI3K, ERK and p38 signalling pathways. 3. Cell migration.	4. HT induced HO-1 mRNA expression in VECs. 5. HT induction of HO-1 and NRF-2 involves PI3K and ERK pathways, but not p38 the pathway. 6. HT increased VECs migration rate.
6.	Zrelli et al. 2011 [37]	To study the effect of HT on proliferation and protection against oxidative stress-induced damage in VECs.	Subject Porcine VECs. Treatment groups HT at 10, 30, 50 and 100 μM concentrations.	1. Cell proliferation, migration and protection against hydrogen peroxide (H_2_O_2_). 2. Phosphorylation of Akt, p38, and ERK1/2 kinase. 3. Cell proliferation and protection against H_2_O_2_ following inhibition of Akt, p38, and ERK1/2 pathways. 4. Nuclear NRF-2 protein levels following inhibition of Akt, p38, and ERK1/2 pathways. 5. Cell proliferation, migration and protection against H_2_O_2_ following inhibition of NRF-2. 6. mRNA and protein levels of HO-1 following inhibition of NRF-2. 7. Cell proliferation, migration and protection against H_2_O_2_ following inhibition of HO-1.	1. HT enhanced cell, migration and maintained VECs viability following H_2_O_2_ exposure. 2. 50 µM HT phosphorylate Akt, p38, and ERK1/2 kinase. 3. Inhibition of Akt and ERK1/2, but not p38 impedes HT-induced cell proliferation and protection against H_2_O_2_. 4. Inhibition of Akt, p38, and ERK1/2 impedes HT-induced elevation of nuclear NRF-2 protein level. 5. Inhibition of NRF-2 impedes HT-induced cell proliferation, migration and protection against H_2_O_2_. 6. Inhibition of NRF-2 impedes HT-induced elevation of HO-1 mRNA and protein levels. 7. Inhibition of HO-1 impedes HT-induced cell proliferation, migration and protection against H_2_O_2_.
7.	Avola et al. 2018 [38]	To investigate the effect of hydroxytyrosol on the irradiated light-emitting-diode-generated blue light (LED-BL) of human dermal fibroblasts (HDFs) and human epidermal keratinocytes (HEKs).	Subject HEKs (NCTC 2544) or HDFs irradiated with LED-BL. Treatment groups HT at 10, 25 and 50 µg/mL concentrations.	1. Cell viability. 2. Reactive oxygen species (ROS) production. 3. DNA damage. 4. Gene and protein expression of MMP-1, MMP-12, collagen type I, and proliferating cell nuclear antigen (PCNA).	1. HT maintains both HEKs and HDFs viability following LED-BL irradiation. 2. HT reduced LED-BL-induced ROS production in both HEKs and HDFs. 3. HT reduced LED-BL-induced DNA damage in both HEKs and HDFs. 4. HT reverses LED-BL-induced increase in MMP-1, MMP-12 and PCNA, as well as the reduction in collagen I gene and protein levels in both HEKs and HDFs.
8.	Meschini et al. 2018 [39]	To investigate the effects of olive vegetation waste (OVW) on lipopolysaccharide (LPS)-stimulated human THP-1 monocytes.	Subject Human THP-1 monocytes stimulated by LPS. Treatment groups OVW treated with immobilised tyrosinase (OVW-1), OVW treated with native tyrosinase (OVW-2), non-treated OVW (OVW-3).	1. Compound content, particularly of HT. 2. Antioxidant activity. 3. DNA damage. 4. Cell cytotoxicity. 5. Cell apoptosis. 6. Cell proliferation. 7. Cell autophagy. 8. Protein expression of high mobility group box 1 (HMGB1) danger signal, granzyme B, IL-6, IL-1β and TNF-α.	1. HT content from highest to smallest were OVW-1, OVW-2 and OVW-3. 2. Antioxidant activity from highest to smallest were OVW-1, OVW-2 and OVW-3. 3. OVW-3 and OVW-1 reduced DNA damage with OVW-1 at 25 μg/mL showing the greatest reduction. 4. Cell viability was not affected by up to 50 μg/mL OVW-1. 5. OVW-1 at 50 µg/mL and 100 µg/mL induced apoptosis after 24 h and 48 h treatment. 6. Cell proliferation started to reduce at 50 µg/mL OVW-1. 7. OVW-1 at 25 µM promote cell autophagy. 8. OVW-1 inhibited the LPS-induced production of HMGB1, granzyme B, IL-6, IL-1β and TNF-α.
9.	Guo et al. 2010 [40]	To investigate the effect of HT towards UVB-induced DNA damage in a human skin keratinocyte cell line, HaCaT	Subject HaCaT induced with UVB. Treatment groups HT: 0, 25, 50 and 100 µM	1. DNA damage. 2. ROS production. 3. Oxidative stress. 4. Phosphorylation of NF-κB. 5. Phosphorylation of p53.	1. HT suppresses UVB-induced DNA damage. 2. HT suppresses UVB-induced ROS formation. 3. HT suppresses UVB-induced oxidative stress. 4. HT attenuates UVB-mediated NF-κB phosphorylation. 5. HT attenuates UVB-mediated p53 phosphorylation.
10.	Jeon and Choi 2018 [41]	To evaluate the anti-inflammatory and antiaging effects of HT via UVA-induced aging model in HDFs.	Subject HDFs irradiated with UVA. Treatment groups HT: 5, 10, 20, 30 µM	1. Cell viability. 2. Ratio of aged cells. 3. Gene expression of MMP-1, MMP-3, IL-1β, IL-6 and IL-8.	1. Cell viability was not affected up to 30 µM of HT. 2. HT decreased UVA-induced aged cells ratio. 3. HT decreased UVA-induced gene expression of MMPs, IL-1β, IL-6 and IL-8.
11.	Ghalandari et al. 2018 [42]	To investigate the antimicrobial effect of HT, HT acetate (HTA) and HT oleate (HTO) on *Staphylococcus aureus* and *Staphylococcus epidermidis*.	Subject *Staphylococcus aureus* or *Staphylococcus epidermidis* Treatment groups HT, HTA or HTO at 3.125, 6.25, 12.5, 25, 50 and 100 mg/mL each.	1. Minimum inhibitory concentration (MIC) for *S. aureus*. 2. Minimum bactericidal concentration (MBC) for *S. aureus*. 3. MIC for *S. epidermidis*. 4. MBC for *S. epidermidis*.	1. MIC were 3.125, 12.5 and 25 mg/mL for HT, HTA and HTO, respectively. 2. MBC were 6.25, 25, 50 mg/mL for HT, HTA and HTO, respectively. 3. MIC were 6.25, 12.5, 50 mg/mL for HT, HTA and HTO, respectively. 4. MBC were 12.5, 25 and 100 mg/mL for HT, HTA and HTO, respectively.
12.	Medina-Martinez et al. 2016 [43]	To determine the antimicrobial activity of HT towards the growth of several bacteria strains	Subject 8 g-negative bacteria, namely, *Erwinia carotovora, Klebsiella pneumonia, Pseudomonas aeruginosa, Yersinia enterocolitica, Salmonella thypimurium, Aeromonas hydrophila, Shigella sonnei, Escherichia coli* and 4 g-positive bacteria, namely, *Pediococcus acidilactici, Kocuria rhizophila, Listeria monocytogenes*, and *S. aureus*. Further experiment with 4 strains of *E. coli*, namely, CECT 4972, CECT 516, CECT 553 and LFMFP 679. Treatment groups HT: 200, 400 and 1000 μg/mL	1. MIC for the 12 test bacteria. 2. Growth kinetic for the four strains of *E. coli*. 3. HT stability.	1. MIC values were mostly equal or higher to 1000 μg/mL, except for *S. aureus* and *E. coli* which showed a MIC of 400 μg/mL. 2. HT at 200 and 400 reduced growth rate of all strains. HT at 1000 µg/mL inhibits the growth of all strains. 3. *E. coli* strains partly reduced HT levels supplemented to the culture media, but not significantly.
13.	Crisante et al. 2015 [44]	To analyse the antioxidant and antibacterial activity of HT-based polyacrylate (HT-pAc) on *S*. *epidermidis*	Subject *Staphylococcus epidermidis* Treatment groups HT-pAc with concentrations of 1, 5 and 10 mg/mL	1. Antioxidant activity. 2. MIC for *S. epidermidis*.	1. HT-pAc at 0.80 ± 0.02 mmol was required to achieve half the maximal antioxidative effect. 2. MIC value of HT-pAc for *S. epidermidis* is 2.5 mg/mL.

## References

[1] Kumar S.A., Vignesh S., Yashavarddhan M.H., Kumar S.S. (2017). Wound healing: Current understanding and future prospect. Int. J. Drug Discov..

[2] Weledji E.P. (2017). Perspectives on wound healing. Aust. J. Surg..

[3] Milne K., Penn-Barwell J. (2020). Classification and management of acute wounds and open fractures. Surg. Oxf..

[4] Dai C., Shih S., Khachemoune A. (2020). Skin substitutes for acute and chronic wound healing: An updated review. J. Dermatol. Treat..

[5] Frykberg R.G., Banks J. (2015). Challenges in the treatment of chronic wounds. Adv. Wound Care.

[6] Martin P., Nunan R. (2015). Cellular and molecular mechanisms of repair in acute and chronic wound healing. Br. J. Dermatol..

[7] Etulain J. (2018). Platelets in wound healing and regenerative medicine. Platelets.

[8] Larouche J., Sheoran S., Maruyama K., Martino M.M. (2018). Immune regulation of skin wound healing: Mechanisms and novel therapeutic targets. Adv. Wound Care.

[9] Stunova A., Vistejnova L. (2018). Dermal fibroblasts—a heterogeneous population with regulatory function in wound healing. Cytokine Growth Factor Rev..

[10] Sorg H., Tilkorn D.J., Hager S., Hauser J., Mirastschijski U. (2017). Skin wound healing: An update on the current knowledge and concepts. Eur. Surg. Res..

[11] Landen N.X., Li D., Stahle M. (2016). Transition from inflammation to proliferation: A critical step during wound healing. Cell. Mol. Life Sci..

[12] Caley M.P., Martins V.L.C., O’Toole E.A. (2015). Metalloproteinases and wound healing. Adv. Wound Care.

[13] Wiegand C., Schonfelder U., Abel M., Ruth P., Kaatz M., Hipler U.C. (2010). Protease and pro-inflammatory cytokine concentrations are elevated in chronic compared to acute wounds and can be modulated by collagen type I in vitro. Arch. Dermatol. Res..

[14] Lawrence T., Fong C. (2010). The resolution of inflammation: Anti-inflammatory roles for NF-κB. Int. J. Biochem. Cell Biol..

[15] Fingleton B. (2017). Matrix metalloproteinases as regulators of inflammatory processes. Biochim. Biophys. Acta Mol. Cell Res..

[16] Krzyszczyk P., Schloss R., Palmer A., Berthiaume F. (2018). The role of macrophages in acute and chronic wound healing and interventions to promote pro-wound healing phenotypes. Front. Physiol..

[17] Schreml S., Szeimies R.M., Prantl L., Karrer S., Landthaler M., Babilas P. (2010). Oxygen in acute and chronic wound healing. Br. J. Dermatol..

[18] Almeida G.C.M., Santos M.M.D., Lima N.G.M., Cidral T.A., Melo M.C.N., Lima K.C. (2014). Prevalence and factors associated with wound colonization by staphylococcus spp. and staphylococcus aureus in hospitalized patients in inland northeastern brazil: A cross-sectional study. BMC Infect. Dis..

[19] McGovern N.N., Cowburn A.S., Porter L., Walmsley S.R., Summers C., Thompson A.A., Anwar S., Willcocks L.C., Whyte M.K., Condliffe A.M. (2011). Hypoxia selectively inhibits respiratory burst activity and killing of staphylococcus aureus in human neutrophils. J. Immunol..

[20] Schaible B., McClean S., Selfridge A., Broquet A., Asehnoune K., Taylor C.T., Schaffer K. (2013). Hypoxia modulates infection of epithelial cells by pseudomonas aeruginosa. PLoS ONE.

[21] Grant S.S., Hung D.T. (2013). Persistent bacterial infections, antibiotic tolerance, and the oxidative stress response. Virulence.

[22] Omara S.T. (2017). MIC and MBC of honey and gold nanoparticles against methicillin-resistant (MRSA) and vancomycin-resistant (VRSA) coagulase-positive s. aureus isolated from contagious bovine clinical mastitis. J. Genet. Eng. Biotechnol..

[23] Kurahashi T., Fujii J. (2015). Roles of antioxidant enzymes in wound healing. J. Dev. Biol..

[24] Dunnil C., Patton T., Brennan J., Barrett J., Dryden M., Cooke J., Leaper D., Georgopoulos N.T. (2015). Reactive oxygen species (ROS) and wound healing: The functional role of ROS and emerging ROS-modulating technologies for augmentation of the healing process. Int. Wound J..

[25] Espinosa-Diez C., Miguel V., Mennerich D., Kietzmann T., Sanchez-Perez P., Cadenas S., Lamas S. (2015). Antioxidant responses and cellular adjustments to oxidative stress. Redox Biol..

[26] Jindam A., Yerra V.G., Kumar A. (2017). Nrf2: A promising trove for diabetic wound healing. Ann. Transl. Med..

[27] Szabo I.L., Kenyeres A., Szegedi A., Szollosi A.G. (2018). Heme oxygenase and the skin in health and disease. Curr. Pharm. Des..

[28] Li B., Wang J.H. (2011). Fibroblasts and myofibroblasts in wound healing: Force generation and measurement. J. Tissue Viability.

[29] Pastar I., Stojadinovic O., Tomic-Canic M. (2008). Role of keratinocytes in healing of chronic wounds. Surg. Technol. Int..

[30] Sorg H., Tilkorn D.J., Mirastschijski U., Hauser J., Kraemer R. (2018). Pantha rei: Neovascularization, angiogenesis and nutritive perfusion in wound healing. Eur. Surg. Res..

[31] Erdogan I., Bayraktar O., Uslu M.E., Tuncel O. (2017). Wound healing effects of various fractions of olive leaf extract (OLE) on mouse fibroblasts. Rom. Biotechnol. Lett..

[32] Cerezo A.B., Labrador M., Gutiérrez A., Hornedo-Ortega R., Troncoso A.M., Garcia-Parrilla M.C. (2019). Anti-VEGF signalling mechanism in HUVECs by melatonin, serotonin, hydroxytyrosol and other bioactive compounds. Nutrients.

[33] Calabriso N., Gnoni A., Stanca E., Cavallo A., Damiano F., Siculella L., Carluccio M.A. (2018). Hydroxytyrosol ameliorates endothelial function under inflammatory conditions by preventing mitochondrial dysfunction. Oxid. Med. Cell. Longev..

[34] Lopez S., Paz S.M., Lucas R., Bermudez B., Abia R., Morales J.C., Muriana F.J.G. (2017). Effect of metabolites of hydroxytyrosol on protection against oxidative stress and inflammation in human endothelial cells. J. Funct. Foods.

[35] Cheng Y., Qu Z., Fu X., Jiang Q., Fei J. (2017). Hydroxytyrosol contributes to cell proliferation and inhibits apoptosis in pulsed electromagnetic fields treated human umbilical vein endothelial cells in vitro. Mol. Med. Rep..

[36] Zrelli H., Kusunoki M., Miyazaki H. (2015). Role of hydroxytyrosol-dependent regulation of HO-1 expression in promoting wound healing of vascular endothelial cells via Nrf2 de novo synthesis and stabilization. Phytother. Res..

[37] Zrelli H., Matsuoka M., Kitazaki S., Araki M., Kusunoki M., Zarrouk M., Miyazaki H. (2011). Hydroxytyrosol induces proliferation and cytoprotection against oxidative injury in vascular endothelial cells: Role of Nrf2 activation and HO-1 induction. J. Agric. Food Chem..

[38] Avola R., Graziano A.C.E., Pannuzzo G., Bonina F., Cardile V. (2018). Hydroxytyrosol from olive fruits prevents blue-light-induced damage in human keratinocytes and fibroblasts. J. Cell. Physiol..

[39] Meschini R., D’Eliseo D., Filippi S., Bertini L., Bizzarri B.M., Botta L., Saladino R., Velotti F. (2018). Tyrosinase-treated hydroxytyrosol-enriched olive vegetation waste with increased antioxidant activity promotes autophagy and inhibits the inflammatory response in human THP-1 monocytes. J. Agric. Food Chem..

[40] Guo W., An Y., Jiang L., Geng C., Zhong L. (2010). The protective effects of hydroxytyrosol against UVB-induced DNA damage in HaCaT cells. Phytother. Res..

[41] Jeon S., Choi M. (2018). Anti-inflammatory and anti-aging effects of hydroxytyrosol on human dermal fibroblasts (HDFs). Biomed. Dermatol..

[42] Ghalandari M., Naghmachi M., Oliverio M., Nardi M., Shirazi H.R.G., Eilami O. (2018). Antimicrobial effect of hydroxytyrosol, hydroxytyrosol acetate and hydroxytyrosol oleate on staphylococcus aureus and staphylococcus epidermidis. Electron. J. Gen. Med..

[43] Medina-Martinez M.S., Truchado P., Castro-Ibanez I., Allende A. (2015). Antimicrobial activity of hydroxytyrosol: A current controversy. Biosci. Biotechnol. Biochem..

[44] Crisante F., Taresco V., Donelli G., Vuotto C., Martinelli A., D’Illario L., Pietrelli L., Francolini I., Piozzi A. (2015). Antioxidant hydroxytyrosol-based polyacrylate with antimicrobial and antiadhesive activity versus staphylococcus epidermidis. Adv. Microbiol. Infect. Dis. Public Health.

[45] Athanasiou A., Karkambounas S., Batistatou A., Lykoudis E., Katsaraki A., Kartsiouni T., Papalois A., Evangelou A. (2007). The effect of pulsed electromagnetic fields on secondary skin wound healing: An experimental study. Bioelectromagnetics.

[46] Kolluru G.K., Bir S.C., Kevil C.G. (2012). Endothelial dysfunction and diabetes: Effects on angiogenesis, vascular remodeling, and wound healing. Int. J. Vasc. Med..

[47] Cano S.M., Lancel S., Boulanger E., Neviere R. (2018). Targeting oxidative stress and mitochondrial dysfunction in the treatment of impaired wound healing: A systematic review. Antioxidants.

[48] Sgonc R., Gruber J. (2013). Age-related aspects of cutaneous wound healing: A mini-review. Gerontology.

[49] Cole M.A., Quan T., Voorhees J.J., Fisher G.J. (2018). Extracellular matrix regulation of fibroblast function: Redefining our perspective on skin aging. J. Cell Commun. Signal..

[50] Basso F.G., Pansani T.N., Turrioni A.P.S., Soares D.G., Costa C.A.D.S., Hebling J. (2016). Tumor necrosis factor-alpha and interleukins IL-1ß, IL-6 and IL-8 impair in vitro migration and induce apoptosis of gingival fibroblasts and epithelial cells, delaying wound healing. J. Periodontol..

[51] Parameswaran N., Patial S. (2010). Tumor necrosis factor-α signaling in macrophages. Crit. Rev. Eukaryot. Gene Expr..

[52] Schöttker B., Brenner H., Jansen E.H.J.M., Gardiner J., Paesey A., Kubinova R., Pajak A., Topor-Madry R., Tamosiunas A., Saum K.U. (2015). Evidence for the free radical/oxidative stress theory of ageing from the CHANCES consortium: A meta-analysis of individual participant data. BMC Med..

